# The application of targeted nanopore sequencing for the identification of pathogens and resistance genes in lower respiratory tract infections

**DOI:** 10.3389/fmicb.2022.1065159

**Published:** 2022-12-22

**Authors:** Hongying Zhang, Meng Wang, Ximei Han, Ting Wang, Yanjuan Lei, Yu Rao, Peisong Xu, Yunfei Wang, Hongcang Gu

**Affiliations:** ^1^Department of Pulmonary Medicine, Fuzhou Pulmonary Hospital of Fujian, Fuzhou, China; ^2^Institute of Health Education, Hangzhou Center for Disease Control and Prevention, Hangzhou, China; ^3^Department of Medicine, Zhejiang ShengTing Biotech Co., Ltd., Hangzhou, China; ^4^Institute of Health and Medical Technology, Hefei Institutes of Physical Science, Chinese Academy of Sciences, Hefei, China; ^5^Graduate School, University of Science and Technology of China, Hefei, China

**Keywords:** pathogen detection, nanopore sequencing, respiratory system, species, resistance genes

## Abstract

**Objectives:**

Lower respiratory tract infections (LRTIs) are one of the causes of mortality among infectious diseases. Microbial cultures commonly used in clinical practice are time-consuming, have poor sensitivity to unculturable and polymicrobial patterns, and are inadequate to guide timely and accurate antibiotic therapy. We investigated the feasibility of targeted nanopore sequencing (TNPseq) for the identification of pathogen and antimicrobial resistance (AMR) genes across suspected patients with LRTIs. TNPseq is a novel approach, which was improved based on nanopore sequencing for the identification of bacterial and fungal infections of clinical relevance.

**Methods:**

This prospective study recruited 146 patients suspected of having LRTIs and with a median age of 61 years. The potential pathogens in these patients were detected by both TNPseq and the traditional culture workups. We compared the performance between the two methods among 146 LRTIs-related specimens. AMR genes were also detected by TNPseq to prompt the proper utilization of antibiotics.

**Results:**

At least one pathogen was detected in 133 (91.1%) samples by TNPseq, but only 37 (25.3%) samples contained positive isolates among 146 cultured specimens. TNPseq possessed higher sensitivity than the conventional culture method (91.1 vs. 25.3%, P < 0.001) in identifying pathogens. It detected more samples with bacterial infections (P < 0.001) and mixed infections (P < 0.001) compared with the clinical culture tests. The most frequent AMR gene identified by TNPseq was *bla*_TEM_ (n = 29), followed by *bla*_SHV_ (n = 4), *bla*_KPC_ (n = 2), *bla*_CTX−M_ (n = 2), and *mecA* (n = 2). Furthermore, TNPseq discovered five possible multi-drug resistance specimens.

**Conclusion:**

TNPseq is efficient to identify pathogens early, thus assisting physicians to conduct timely and precise treatment for patients with suspected LRTIs.

## Introduction

Lower respiratory tract infections (LRTIs) are one of the most lethal infectious diseases, the majority of which develop into pneumonia, bronchitis, and bronchiolitis, contributing to the increasing number of mortality and morbidity worldwide, particularly among senior citizens in low-income regions (Feldman and Shaddock, 2019; Troeger et al., 2019; Collaborators, 2020). They give rise to 3.5 million deaths and 79 million cases of disabilities annually (Shao et al., 2022). A good deal of pathogens are responsible for infection of the lower respiratory tract such as bacteria, fungi, virus, and atypical pathogens (Man et al., 2017; Chen et al., 2021). More severe diseases can occur in the lungs after an invasion of secondary bacterial pneumonia (Keshavarz et al., 2018).

Traditional culture has long been the gold standard for detecting bacterial or fungal respiratory infections (Gu et al., 2021). However, there are still some pathogens that have not yet been detected. Due to the low-nutrient environment and low bacterial burden of the respiratory tract, most infectious agents are difficult or even virtually impossible to recognize using routine culture (Yatera et al., 2021). In addition, the cultural conditions for these pathogens are rigorous and time-consuming. Immunological methods and polymerase chain reaction (PCR) are also used for laboratory diagnosis of clinical microbes; nonetheless, only a narrowly presupposed group of microorganisms can be detected and identified (Hong et al., 2020). In this case, patients with severe LRTIs were broad-spectrum treated according to the previous experience of physicians. At the same time, antibiotic resistance, caused by the inappropriate use of antimicrobial agents, is likely to cause ineffective treatment, which increases the risk of opportunistic infections, hospital mortality rates, and healthcare costs (Boolchandani et al., 2019). Hence, timely etiological identification is crucial for accurate treatment and early recovery.

Advances in sequencing technology facilitate its application in clinical practice for pathogen identification. Next-generation sequencing (NGS), a culture-independent molecular method, possesses hypothesis-free detection capacity to cover nearly all types of microorganisms (Jun et al., 2021). Nevertheless, the widespread availability of conventional NGS analysis is influenced by host DNA, symbiotic microflora-colonized native positions, and potential contamination by the environment (Gu et al., 2019). Furthermore, its high cost and slow turnaround time concern users. Nanopore sequencing is a new-generation sequencing technology that could alleviate the drawbacks of NGS and PCR (Charalampous et al., 2019; Fu et al., 2022). In recent years, it has emerged to identify pathogenic microbes in infectious and non-infectious diseases (Petersen et al., 2019; Wang et al., 2020). This advance is attributed to its fast turnaround time, long read length, and pathogen enrichment (Deamer et al., 2016; Ciuffreda et al., 2021), which is free from the interference of microflora colonization and host depletion. In addition to pathogen detection, nanopore sequencing can predict antimicrobial resistance (AMR) genes, to speculate the phenotype of resistance (Petersen et al., 2019). However, the characterization of AMR genes with nanopore sequencing is scarce in clinical practice.

In the present study, we compared the clinical effectiveness of targeted nanopore sequencing (TNPseq) with culture, in the pathogenic diagnosis of LRTIs. In addition, the antibiotic resistance genes of pathogens were predicted in patients with LRTIs *via* TNPseq.

## Materials and methods

### Patient recruitment and specimen collection

Between January and April 2022, 146 patients, including 106 male patients and 40 female patients, were enrolled in the Fuzhou Pulmonary Hospital of Fujian in China for this study. Except for three children, the age range was 16–87 years. These patients were suspected of having LTRIs grounded on clinical symptoms and physical signs, blood biochemistry, imaging features of the chest, and laboratory results. One hundred and forty-six specimens were collected in dedicated sterile tubes after patients had given their written informed consent. All specimens were aseptically divided into two equal parts without dilution. One part was sent to the microbiological laboratory in Fuzhou Pulmonary Hospital for bacterial and fungal culture. The other part, under ice bags or dry ice, was promptly sent to Hangzhou ShengTing Biotech Co. Ltd. (Hangzhou, China) for TNPseq using cold chain logistics.

### Clinical microbiology trials

All samples were routinely analyzed according to *The National Clinical Test Regulation of Operation* for the detection of pathogens at clinical microbiology laboratories (Shang et al., 2014). Bronchoalveolar lavage fluid (BALF) was concentrated using centrifugation at 3,000 rpm for 10 min and inoculated onto Columbia blood agar, chocolate agar, and MacConkey agar plates. Then, these plates were incubated at 35 ± 1°C overnight for bacterial culture in an incubator with 5% CO_2_ or in anaerobic bags. In order to culture fungi, Sabouraud's dextrose agar was used and incubated at 30°C for 48–72 h. Pathogen identification was performed using a BD Phoenix™ M50 Automated Microbiology System (BD, Inc., USA) and a VITEK^®^ Mass Spectrometry Microbial Identification System (bioMérieux, Inc., France). Viral cultures were not carried out due to limitations of the biosafety facilities.

### DNA extraction and amplification

The samples were initially centrifuged, digested with proteinase K and lysozyme, and adequately ground using zirconia grinding beads and standard laboratory practices, according to different specimen sources, to improve the capacity of pathogen detection. The nucleic acid of ground samples was extracted with magnetic beads using the QIAamp DNA Microbiome Kit (Cat. No.51707, Qiagen, Hilden, Germany) and following the manufacturer's protocol. Blank EB buffer was concurrently used as the negative control for the extraction of nucleic acid. The concentration of extracted DNA was detected using an Invitrogen Qubit 4 Fluorometer. Then, PCR amplification was carried out using the universal primers 27F/1492R for bacterial 16S rRNA gene detection and ITS1/4 for fungal IST1/2 gene detection (Chan et al., 2020; Jun et al., 2021). PCR amplification was performed on an ABI 2720 Thermal Cycler (Cat. No. 435659; ABI, California, USA) with conditions as follows: initiation at 95°C × 3 min, 25 cycles of 95°C × 30 s/62°C × 60 s/72°C × 60 s, and a final extension step of 72°C × 3 min. The products of PCR amplification were purified and then quantified using Qubit 4 and agarose gel electrophoresis for subsequent library preparation and nanopore sequencing.

### Library preparation and nanopore sequencing

Nanopore barcode PCR was performed with the aforementioned products according to the PCR Barcoding Expansion Pack 1-96 kit (EXP-PBC096). Library pooling was accomplished utilizing ~50 ng of gDNA with the VAHTS Universal DNA Library Prep Kit (Cat. No. ND607-01, Nanjing Vazyme Biotech, Nanjing, CN). Subsequently, nanopore library preparation was constructed using a ligation sequencing kit (Cat. No. SQK-LSK109, Oxford Nanopore Technologies, Oxford, UK) as per the manufacturer's instructions. Ultimately, ~100 ng of the pooled library was loaded into a nanopore flow cell (R9.4.1) after chip priming, and sequenced utilizing the GridION platform. MinKNOW version 2.0 was used for outputting base-calling data. Then, barcode demultiplexing was conducted using Porechop (v. 0.2.4).

### Identification of pathogen and resistant genetype

Real-time identification of pathogens was performed *via* the 16S workflow and the “What's in my Pot?” (WIMP) workflow, using the EPI2ME platform (version 3.2.2, ONT, Oxford, UK). Furthermore, the EPI2ME platform offered bacterial classification *via* the 16S workflow as well as identification of fungi, bacteria, viruses, or archaea *via* the WIMP workflow. Reads under 200 bp were filtered, and the remaining reads were aligned to all targets and potentially etiological agents using the National Center for Biotechnology Information (NCBI) basic local alignment search tool (BLAST), or reanalyzed using GAIA 2.0 (Chan et al., 2020; Fu et al., 2022). Pathogens were classified at the species level based on the percentage of coverage and identity. Generally, the top 10 microorganisms, ranked by aligned reads and with a relative abundance score of >0.5% were classified as pathogens and subjected to further evaluation. Potential pathogen(s) were reported if the number of reads accounted for ≥1% of microbial reads and if the WIMP alignment score was ≥20 (Charalampous et al., 2018). *Mycobacterium tuberculosis* was considered positive when at least one read was mapped to either the species or genus level (Miao et al., 2018). AMR genes detected by TNPseq were predicted using the Comprehensive Antibiotic Resistance Database (CARD) or the ResFinder database with default alignment settings (≥80% identity over ≥60% of the length of the target gene) (Yonkus et al., 2022).

### Statistical analyses

Continuous variables are presented as mean ± standard deviation. Percentages are used to describe individual microbial detection rates in patients. The differences between culture and TNPseq were assessed using the chi-square test *via* IBM SPSS Statistics 24.0 software (IBM Corp, Armonk, NY, USA). Statistical differences were considered significant if the *P*-value was < 0.01. Heatmaps and Venn diagrams were plotted on https://www.bioinformatics.com.cn (last accessed on 30 July 2022), an online platform for visualization.

## Results

### Patient demographics

We enrolled 146 patients with suspected LRTIs. The median age was 61 years, and 106 of the 146 patients were male. Samples were collected, including 122 BALF, seven sputa, eight blood, six puncture fluids, and three transbronchial lung biopsies (Table 1). Of these 146 patients, there were 37 cases of microbial growth from culture trials, including eight mixed infections. In this research, mixed infection was defined as different species infecting the same individual. As for TNPseq, 133 cases were identified at the species level carrying a potential pathogen, and there were 48 cases in which only one species was detected. In addition, AMR genes were detected in 37 patients.

**Table 1:** Cohort characteristics.

**Characteristics**	**Data**
Median age (Years)	61
**Gender**, ***n*** **(%)**	
Male	106 (72.6%)
Female	40 (27.4%)
C-reactive protein median (mg/L, IQR)	21.06 (7.17, 68.04)
Procalcitonin median (μg/L, IQR)	0.12 (0.06, 0.79)
Neutrophil count (10^9^/L, mean ± SD)	9.53
White blood cell count (10^9^/L, mean ± SD)	9.27
**Sample type**, ***n*** **(%)**	
BALF	122 (83.6%)
Sputum	7 (4.8%)
Blood	8 (5.5%)
Puncture fluid	6 (4.1%)
Transbronchial lung biopsy	3 (2.0%)
**Culture**, ***n*** **(%)**	
Negative	109 (74.7%)
Positive	37 (25.3%)
**TNPseq**, ***n*** **(%)**	
Positive	133 (91.1%)
Negative	13 (8.9%)
Only one pathogen, *n* (%)	48 (32.9%)
Antibiotic resistance genes, *n* (%)	36 (24.7%)

BALF, bronchoalveolar lavage fluid.

### Comparison of TNPseq with conventional culture

**Figure 1:**
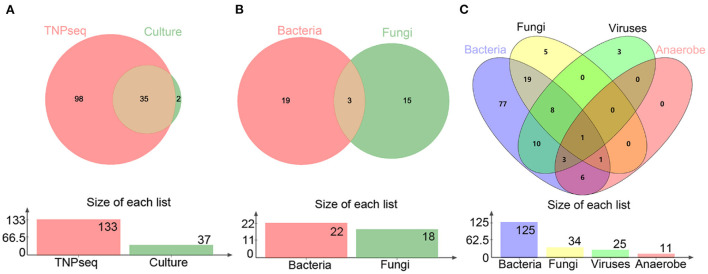
Venn diagrams of positive specimens *via* TNPseq and culture. **(A)** The number of sets for positive TNPseq and culture specimens. **(B)** Bacteria and fungi identified by culture. **(C)** Bacteria, fungi, viruses, and anaerobes identified by TNPseq.

In the present study, the common opportunistic pathogens or microbes harming human health in clinical practice were defined as positive when detected by TNPseq. We identified 133 of 146 (91.1%) samples that provide refuge for pathogens, including 125 bacteria, 34 fungi, 25 viruses, 11 anaerobic bacteria, and 85 mixed infections using TNPseq. The details of mixed infections under different classifications among the aforementioned microbes are shown in Figure 1. A total of 37 of 146 (25.3%) samples harboring 22 bacteria, 18 fungi, and eight mixed infections were detected by routine culture. The sensitivity was significantly increased by 58.9% in TNPseq compared with culture (91.1 vs. 25.3%; *P* < 0.001). A comparison of TNPseq and routine culture of 146 samples is shown in Table 2. Out of these results, TNPseq successfully detected possible etiological agents from 98 of the 109 culture-negative cases (89.9%). In addition, the positive rate of bacteria was higher than that of fungi. The sensitivity of TNPseq was superior to culture in detecting anaerobic bacteria, viruses, and mixed infections. This is imperative for the precision treatment of patients with suspected LRTIs, as polymicrobial infections were not easily perceived using clinical standard workups such as anaerobic culture, viruses were often not detected, or specific classification was lacking during routine clinical culture. The cost of TNPseq was ~1,000 RMB. The time span from test initiation to the delivery of test results for TNPseq was ~16–17 h, whereas the time span for the culture test was at least 24–72 h.

**Table 2:** Effectiveness of routine microbial trails in comparison with targeted nanopore sequencing.

**Pathogen**	**TNPseq positive**	**Culture**				***P*-value**
		**Culture positive**	**TNPseq positive** ^#^	**Culture negative**	**TNPseq positive** ^*^	
Total	133 (91.1%)	37	35 (94.6%)	109	98 (89.9%)	< 0.001
Anaerobe	11 (7.5%)	0	0	146	11 (7.5%)	0.001
Bacteria	125 (84.9%)	22	21 (95.4%)	124	104 (83.8%)	< 0.001
Fungi	34 (20.5%)	18	11 (61.1%)	128	23 (17.9%)	< 0.001
Viruses	25 (16.4%)	0	0	146	25 (17.1%)	< 0.001
Mixed infection	85 (54.1%)	8	8 (100%)	138	77 (55.8%)	< 0.001

Mixed infection was defined as different species infecting the same individual. The percentage labeled with ^**#**^ was obtained from TNPseq and culture double positive/double positive + single culture positive; the percentage labeled with

^*^ was obtained from TNPseq positive/culture negative.

As for consistency, 35 out of 37 (94.6%) positive results were detected from the same patients by TNPseq and culture (Figure 1), among 27 (73.0%) cases of which the TNPseq results were entirely consistent for clinical microbiology workups. In other words, microbial strains were present in two samples, but the coincident microbe was absent in TNPseq of the same samples. The results from eight double positive samples analyzed using culture and TNPseq were paradoxical. Ten discrepant cases are listed in Table 3. This incompatibility might be caused by the inadequately lysed fungal cell wall through lysozyme or misidentified EPI2ME.

**Table 3:** Discrepant cases among the TNPseq and culture test.

**Case**	**Gender**	**Age**	**Primary diagnosis**	**Sample**	**TNPseq (No. of reads)**	**Culture**
21T005333	Male	51	Severe pneumonia	BALF	Negative	*C. albicans*
22T0007678	Male	63	Tuberculosis	BALF	Negative	*E. coli*
22T000494	Male	68	Pneumonia	BALF	*C. argentoratense* (5924)	*C. glabrata*
22T0007653	Male	55	Pneumonia	BALF	*H. haemolyticus* (9), *T. denticola* (556)	*C. glabrata*
22T001387	Male	63	AECOPD	BALF	*H. haemolyticus* (490), *E. rhusiopathiae* (3), *C. albicans* (2), *Human gammaherpesvirus* (42)	*C. albicans, Aspergillus*
22T0016635	Male	37	ABPA	BALF	*H. influenzae* (3)	*Aspergillus*
22T0016719	Male	67	LC, pneumonia	BALF	*H. influenzae* (4)	*Aspergillus*
22T003154	Female	78	COPD	BALF	*P. aeruginosa* (3510), *C. striatum* (1973)	*P. aeruginosa, S. maltophilia*
22T005899	Male	63	Abdominal and pulmonary shadow	BALF	*M. intracellulare* (15), *S. pneumoniae* (34), *A. penicillioides* (7)	*M. avium*
22T0007741	Male	75	COP, pneumonia, secondary pulmonary tuberculosis	Blood	*H. influenzae* (2)	*A. baumannii–A. calcoaceticus complex*

AECOPD, acute exacerbation of chronic obstructive pulmonary disease; ABPA, allergic bronchopulmonary aspergillosis; LC, lung carcinoma; PI, pulmonary infection; COPD, chronic obstructive pulmonary disease; COP, cryptogenic organizing pneumonia; TNPseq, targeted nanopore sequencing.

### Microbial population in patients with LRTIs

**Figure 2:**
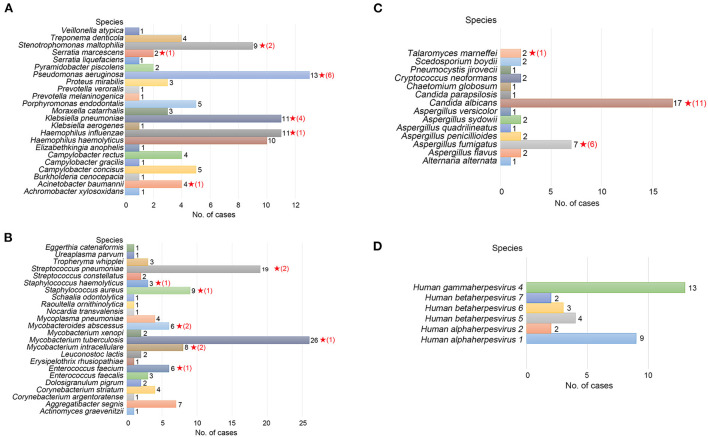
Microbial communities of pathogens at the species level of LRTI specimens. **(A)** Gram-negative bacteria. **(B)** Gram-positive bacteria. **(C)** Fungi. **(D)** Viruses. The red, five-pointed star behind the bar represents species isolated in the clinical culture test, and the number in parentheses represents the number of cases.

For identifying the etiological agent of LRTIs, detection at the species level is essential for early diagnosis and precision therapy, particularly for *Streptococcus* and *Mycobacterium* species. However, NGS analysis is frequently not precise enough to identify pathogens at the species level (Haro et al., 2020). We identified 24 species of gram-negative bacteria, 23 species of gram-positive bacteria, 14 species of fungi, and six species of viruses by TNPseq, which has implications for the diagnosis and treatment of patients. The top five gram-negative bacteria were *Pseudomonas aeruginosa* (*n* = 13), *Haemophilus influenzae* (*n* = 11)*, Klebsiella pneumoniae* (*n* = 11)*, Haemophilus haemolyticus* (*n* = 10), and *Stenotrophomonas maltophilia* (*n* = 9) (Figure 2A). Among 23 detected gram-positive bacteria, *Mycobacterium tuberculosis* (*n* = 26) is superior in number in all specimens, followed by *Streptococcus pneumoniae* (*n* = 19), *Staphylococcus aureus* (*n* = 9), *Mycobacterium intracellulare* (*n* = 8), and *Enterococcus faecium* (*n* = 6) (Figure 2B). Common etiologic microbes of fungal pneumonia were detected, such as *Candida albicans* (*n* = 17), *Aspergillus fumigatus* (*n* = 7), and *Cryptococcus neoformans* (*n* = 2) (Figure 2C). *Human gammaherpesvirus* 4 (*n* = 13), *Human alphaherpesvirus* 1 (*n* = 9), and *Human betaherpesvirus* 5 (*n* = 4) were the top three viral species (Figure 2D). The results of microbial populations in patients with LRTIs, identified *via* TNPseq, conformed to the epidemiology of respiratory infections in China.

### Antibiotic resistance

**Figure 3:**
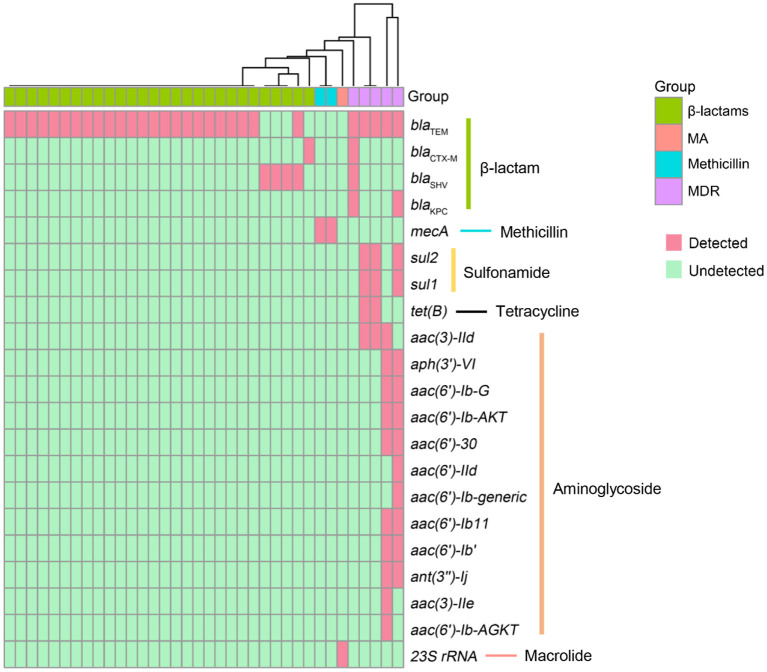
Antimicrobial resistance (AMR) genes presented in LRTI specimens. The rows represent AMR genes and the columns represent specimens. MA, macrolide antibiotics; MDR, multidrug resistance.

Antimicrobial resistance contributes to high mortality and economic costs every year. To assess the antibiotic resistance of patients with LRTIs, we predicted the AMR genes, which can potentially complicate disease treatments and can potentially be transferred to other bacteria with the help of CARD. In the present study, a total of 22 different antimicrobial resistance (AMR) genes were detected among 36 patients (Figure 3). The most prevalent AMR gene in the samples of patients with suspected LRTIs was *bla*_TEM_. The other percentages of AMR genes were affiliated with *bla*_SHV_ (*n* = 3), *mecA* (*n* = 2), *bla*_CTX − M_ (*n* = 1), and 23S rRNA (*n* = 1). Moreover, we identified five specimens carrying multidrug resistance (MDR) genes. The results of the antibiotics susceptibility test of patients in this trial are presented in Supplementary Table 1. The presence or absence of a resistance gene is not fully correlated with the resistance phenotype.

## Discussion

Culture-dependent methods have severe bottlenecks as guides for the etiologic diagnosis of acute critical infection due to time-consumption, narrow-spectrum detection, and susceptibility to the previous history of antibiotic use. The time required for TNPseq was ~16–17 h from test initiation to the delivery of test results, which was less than that for Illumina NGS (~22–24 h) and for the clinical culture test (~24–72 h). A timely and precise method is essential to balance the clinical curative effect and increased mortality, since empiric therapy is not the best solution in the face of severe pulmonary infection (Glimåker et al., 2015; McGill et al., 2016; Charalampous et al., 2018). TNPseq is a strategy of pivotal importance for the early diagnosis of slow-growing pathogens, for instance, *Mycobacterium* and *Aspergillus*, or for seriously infected patients in urgent need of treatment, with its rapid turnaround time and broad coverage of pathogens (Gu et al., 2021). In the current study, we investigated the effectiveness of TNPseq for the identification of pathogens and AMR genes in 146 patients with a high clinical suspicion of LRTIs.

The sensitivity of TNPseq for the cases of suspected LRTIs was significantly higher than that of typical culture workups in our study. The results were consistent with previous reports (Gu et al., 2021; Hoefnagels et al., 2021; Jun et al., 2021; Fu et al., 2022). As for the samples that were only culture positive, two pathogens of possible single infection detected by culture were missed by TNPseq: *C. albicans* and *Escherichia coli*. The first of the two missed cases was a case with severe pneumonia. Clinical microbial testing showed a diagnosis of a single infection by *C. albicans*. In contrast, TNPseq testing did not detect the common pathogen. The upper respiratory commensals were identified, and included *Streptococcus mitis, Stomatococcus mucilaginosus, and Actinomyces odontolyticus*. The other missed case was that of a patient preliminarily diagnosed with tuberculosis; only *E. coli* was detected by the culture test in the BALF specimen. Similarly, microorganisms such as *Haemophilus parahaemolyticus, Streptococcus parasanguinis*, and *Gemella haemolysans* were recognized as microbes colonizing human beings rather than common clinical pathogens. Chan et al. (2020) also reported that the sensitivity for low-abundance microbial specimens was lower in a background of upper respiratory microbiota, and species were likely misidentified by EPI2ME. As for double positive samples *via* culture and TNPseq, *Candida glabrata, C. albicans*, and/or *Aspergillus* were detected in five samples *via* culture, but bacteria such as *C. argentoratense, H. haemolyticus*, and *T. denticola*, were detected *via* TNPseq. We speculate that the thick fungal cell wall or its low biomass in DNA extraction (Bittinger et al., 2014) contributes to the non-detection of these fungi. *S. maltophilia, M. avium*, and *A. baumannii–A. calcoaceticus complex* were detected *via* culture, while TNPseq failed to recognize them in three double positive cases. Verifying microbial taxon with alternative pipelines is advisable to improve misidentification, and the trade-offs are easy to determine, compared with the high sensitivity. Moreover, TNPseq detected likely pathogens from 98 of 109 culture-negative specimens. Thus, TNPseq is an effective method to identify etiologic microorganisms by comparing routine cultures in clinical microbiology laboratories.

The infection-associated microbiota we detected in lower respiratory tract specimens substantially overlap with the microbial population detected in patients with acute respiratory infections in China during 2009–2019 (Li Z. J. et al., 2021). *S. pneumoniae, M. pneumoniae, P. aeruginosa, K. pneumoniae*, and *H. influenzae* were ranked according to the proportion of positive detection in the adults. In addition, more *Mycobacterium* including *M. intracellulare*, were identified in our research using the specific TNPseq method. TNPseq facilitates the detection of slow-growing pathogens and has solved the difficulties of detecting intracellular bacteria to a certain extent. As for the species level of fungi, *A. fumigatus* is the second most common etiological agent after *C. albicans* in the present study. The early diagnosis of Aspergillosis is mandatory for successful treatment outcomes (Ahmad and Khan, 2020). Furthermore, we have detected a high number of polymicrobial infections using TNPseq. Bacterial–fungal co-infection (*n* = 19) was the most frequent co-infection, and bacterial–viral co-infection (*n* = 10) took second place. It is interesting to note that eight cases of polymicrobial infection with bacteria, fungi, and viruses occurred simultaneously. Independent studies documented similar results, in which bacteria–fungi mixed infection was the most common mode of mixed infection (Leclair and Hogan, 2010; Xie et al., 2021; Nebreda-Mayoral et al., 2022). Bacterial–fungal co-infection, often leads to pulmonary fibrosis and contributes to respiratory failure and even premature death. TNPseq is conducive to the early diagnosis and accurate treatment of pathogens, especially slow-growing pathogens, bacterial–fungal co-infection, and viral co-infection, as well as to improving the prognosis of patients with pneumonia.

Antibiotic resistance due to the misuse and abuse of antibiotics is a global challenge to the therapy of infectious diseases. The transmission and persistence of resistance genes among pathogens have led to attentiveness in public health worldwide (Bhullar et al., 2012; Emond-Rheault et al., 2020; Li R. et al., 2021). In early studies, nanopore sequencing was applied to detect AMR genes in different pathogens. The long reading length of nanopore sequencing is a considerable advantage for reducing omissions of base pairs or small fragments of DNA during the process of assembly, which is crucial for the identification of mutational AMR genes (Koren and Phillippy, 2015; Lemon et al., 2017). The *bla*_TEM_ gene (*n* = 29) was the most commonly detected among the β-lactamase-encoding genes, followed by *bla*_SHV_ (*n* = 4), *bla*_KPC_ (*n* = 2), and *bla*_CTX − M_ (*n* = 2). KPC is a multienzyme belonging to class A carbapenemases, which are usually produced by *Enterobacteriaceae*, particularly *K. pneumoniae* and *P. aeruginosa* (Poirel et al., 2010; Liao et al., 2022). In the present study, *K. pneumoniae, H. influenzae*, and *P. mirabilis* were identified in the specimens with the *bla*_KPC_ gene detected by TNPseq; however, the conventional culture showed no evidence of microbial growth. In addition, *bla*_KPC_ was the predominant carbapenem resistance gene associated with multidrug-resistant (MDR) pathogens (Haider et al., 2022; Zheng et al., 2022), which is why specimens containing the *bla*_KPC_ gene clustered in the group of MDR. Among the groups of MDR, aminoglycoside-modifying enzyme (AME)-encoding genes were another example of notable AMR genes. AME-encoding genes are one of the most important resistance genes transferable between gram-negative bacteria, especially in *P. aeruginosa* (Belaynehe et al., 2017). *aac(6*′*)-II, aac (6*′*)-I, ant(2*″*)-I*, and *aph(3*′*)* are the most common AME-encoding genes in *P. aeruginosa* (Ahmed, 2018). We detected 12 AME-encoding genes distributed in two cases of culture-negative specimens. Antibacterial drugs had often been used before sample collection, which may have reduced the culture detection rate. At the same time, *P. aeruginosa, H. influenzae*, and *P. mirabilis* were discovered in these two specimens using TNPseq. Similarly, *aph(3')-VI, aac(6')-Ib-G, aac(6')-IId, aac(6')-Ib', aac(6')-Ib11, ant(3”)-Ij, aac(6')-30, aac(6')-Ib-AGKT, aac(6')-Ib-AKT*, and *aac(3)-IIe* were detected in the specimen in which *P. aeruginosa* was identified. These results implied the crucial role of TNPseq in the identification of pathogens and AMR genes. The application of TNPseq could, to a certain extent, help clinicians make decisions regarding specific antibiotics rather than broad-spectrum antibiotics.

One of the limitations of this study is that only a small number of other sample types, except for BALF, were included in the present research. Future studies of larger sample sizes, including saliva, blood, puncture fluid, and transbronchial lung biopsy, are required to fully explore and compare the performances of TNPseq and conventional culture methods. Moreover, the AMR genes identified by TNPseq did not provide information on susceptibility to antibiotics. It is necessary to further verify the antimicrobial susceptibility test for more patients in the clinical laboratory.

In conclusion, our results underlined the potential value of TNPseq for the rapid identification of etiological agents. From a comprehensive perspective, TNPseq is an effective method for the early identification of pathogens in patients with LRTI. It is vital to treat dangerous acute severe infectious diseases precisely to improve patients' outcomes.

## Data availability statement

The datasets presented in this study can be found in online repositories. The names of the repository/repositories and accession number(s) can be found below: https://www.ncbi.nlm.nih.gov/, PRJNA 902511.

## Ethics statement

The studies involving human participants were reviewed and approved by Ethics Committee of Fuzhou Tuberculosis Prevention Hospital (number 2022-004-01), Fujian Province. The patients/participants provided their written informed consent to participate in this study.

## Author contributions

HZ, MW, YW, and HG conceived, designed, and supervised the study. XH, TW, and YR were responsible for patient recruitment. PX formulated the protocols and performed the experimental implementation process. HZ, MW, YL, and HG analyzed the data and drafted the manuscript. All authors proofread, revised the draft, and agreed to the published version of the manuscript.
