# Supplementary material for: The application of targeted nanopore sequencing for the identification of pathogens and resistance genes in lower respiratory tract infections
Source: Front Microbiol. 2022 Dec 22;13:1065159. doi: 10.3389/fmicb.2022.1065159 (PMC9822541; doi:10.3389/fmicb.2022.1065159)
Supplement: Supplementary file 1 [file Table_1.DOCX]

**Supplementary table 1** The cases detected drug resistance genes by Nanopore sequencing

| Case | Pathogen by culture | Pathogen by Nanopore (No. of reads) | Resistance genes (No. of alignments) | Antimicrobial susceptibility test |
| --- | --- | --- | --- | --- |
| 22T0017531 | *Klebsiella pneumoniae* | *Mycobacterium intracellulare* (117)  *Klebsiella pneumoniae* (151)  *Haemophilus haemolyticus* (45)  *Serratia marcescens* (9)  *Treponema denticola* (9) | blaTEM (28) | S: all tested agents |
| 22T005882 | *Pseudomonas aeruginosa* | *Pseudomonas aeruginosa* (633) | blaTEM (1) | S: all tested agents |
| 22T0007742 | *Candida albicans, Aspergillus fumigatus* | *Mycobacterium tuberculosis* (2)  *Haemophilus influenzae* (246)  *Candida albicans* (7759)  *Aspergillus fumigatus* (2) | blaTEM (1) | S: Flucytosine, Fluconazole, Voriconazole, Amphotericin B, Itraconazole |
| 22T007358 | *Klebsiella pneumoniae* | *Mycobacterium tuberculosis* (130)  *Klebsiella pneumoniae* (20)  *Mycobacterium intracellulare* (20) | blaTEM (14) | Not tested |
| 22T0009278 | *Enterococcus faecalis* | *Porphyromonas endodontalis* (3086)  *Enterococcus faecalis* (31)  *Human betaherpesvirus 5* (16)  *Human gammaherpesvirus 4* (6) | Negative | R: Penicillin  S: Gentamicin, Ampicillin, Vancomycin  I: Linezolid |
| 22T0007741 | *Acinetobacter calcoaceticus-Acinetobacter baumannii complex* | *Haemophilus influenzae* (2) | Negative | S: Ceftazidime, Piperacillin-tazobactam, Meropenem, Amikacin, Gentamicin, Polymyxin, Ciprofloxacin  I: Cefotaxime |
| 22T005306 | *Pseudomonas aeruginosa* | *Pseudomonas aeruginosa* (9364) | Negative | I: Polymyxin, Ciprofloxacin  S: Gentamicin, Amikacin, Imipenem, Meropenem |
| 22T005752 | *Klebsiella pneumoniae*  *Mycobacterium tuberculosis* | *Mycobacterium tuberculosis* (12)  *Klebsiella pneumoniae* (30) | Negative | S: all tested agents |
| 21T010888 | *Pseudomonas aeruginosa* | *Pseudomonas aeruginosa* (960)  *Candida albicans* (5) | Negative | I: Cefepime |
| 21T005578 | *Candida albicans* | *Nocardia transvalensis* (31)  *Candida albicans* (10190) | Negative | S: Flucytosine, Fluconazole, Voriconazole,  Amphotericin B Itraconazole |
| 22T002594 | *Pseudomonas aeruginosa* | *Pseudomonas aeruginosa* (60) | Negative | R: Imipenem, Meropenem  I: Polymyxin |
| 22T000494 | *Candida* [*glabrata*](javascript:;) | *Corynebacterium argentoratense* (5924) | Negative | S: Flucytosine, Fluconazole, Voriconazole,  Amphotericin B Itraconazole |
| 21T007405 | *Serratia marcescens* | *Serratia marcescens* (8)  *Raoultella ornithinolytica* (2) | Negative | R: Tetracycline |
| 22T0016637 | *Streptococcus pneumoniae, Mycobacterium intracellulare* | *Streptococcus pneumoniae* (14)  *Haemophilus influenzae* (209)  *Human alphaherpesvirus* 1 (30)  *Human alphaherpesvirus* 2 (3) | blaTEM (202) | Not tested |
| 22T004595 | *Streptococcus pneumoniae* | *Streptococcus pneumoniae* (940)  *Aggregatibacter segnis* (174)  *Dolosigranulum pigrum* (2)  *Human gammaherpesvirus* 4 (33) | blaTEM (841) | Not tested |
| 22T005899 | *Streptococcus pneumoniae* | *Mycobacterium intracellulare* (15)  *Streptococcus pneumoniae* (34)  *Aspergillus penicillioides* (7)  *Klebsiella pneumoniae* | blaTEM (2) | Not tested |
| 22T005401 | *Cryptococcus neoformans* | *Cryptococcus neoformans* (14)  *Klebsiella pneumoniae*  *Human alphaherpesvirus* 1 | blaSHV (1) | Not tested |
| 22T007041 | *Staphylococcus aureus* | *Mycobacterium tuberculosis* (3)  *Haemophilus haemolyticus* (487)  *Staphylococcus aureus* (12)  *Candida albicans* (6)  *Human gammaherpesvirus* 4 (10) | mecA (8) | Not tested |
| 22T006951 | *Candida albicans* | *Pseudomonas aeruginosa* (3)  *Candida albicans* (5) | blaCTX-M (2） | Not tested |
| 21T003423 | *Klebsiella pneumoniae, Candida albicans* | *Klebsiella pneumoniae* (11722), *Candida albicans* (2782) | blaSHV | Not tested |
| 21T003545 | *Klebsiella pneumoniae, Candida albicans* | *Klebsiella pneumoniae* (16752), *Candida albicans* (3) | blaSHV | Not tested |
| 22T003064 | *Hemophilus influenzae* | *Acinetobacter baumannii* (83)  *Hemophilus influenzae* (10)  *Pseudomonas aeruginosa* (6) | blaTEM | Not tested |
| 22T003646 | *Pseudomonas aeruginosa, Stenotrophomonas maltophilia* | *Corynebacterium striatum* (3249)  *Pseudomonas aeruginosa* (3081)  *Stenotrophomonas maltophilia* (648)  *Achromobacter xylosoxidans* (395)  *Proteus mirabilis* (71)  *Enterococcus faecium* (25) | blaTEM (1) | Not tested |
| 22T003122 | *Streptococcus pneumoniae* | *Streptococcus pneumoniae* (3769) | blaTEM (10) | Not tested |
| 22T0007653 | *Candida glabrata* | *Haemophilus haemolyticus* (9)  *Treponema denticola* (556) | blaTEM(8) | Not tested |
| 22T0010476 | Negative | *Staphylococcus aureus* (355)  *Enterococcus faecium* (4)  *Acinetobacter baumannii* (4)  *Candida albicans* (446)  *Human alphaherpesvirus* 1 (1239)  *Human gammaherpesvirus* 4 (4) | mecA(5) | Not tested |
| 22T0016098 | Negative | *Streptococcus pneumoniae* (122) | blaTEM (19) | Not tested |
| 22T0007747 | Negative | *Mycobacterium tuberculosis* (2)  *Klebsiella pneumoniae* (10) | blaTEM (1) | Not tested |
| 21T008153 | Negative | *Mycobacterium tuberculosis* (5) | blaTEM (1) | Not tested |
| 22T0012664 *^#^* | Negative | *Solobacterium moorei* (14)  *Fusobacterium nucleatum* (6)  *Cutibacterium acnes* (3)  *Acinetobacter guillouiae* (1) | blaTEM (1) | Not tested |
| 22T004805 | Negative | *Haemophilus haemolyticus* (237)  *Moraxella catarrhalis* (33) | blaTEM (140) | Not tested |
| 22T0009080 | Negative | *Mycobacterium intracellulare* (10)  *Haemophilus influenzae* (365)  *Tropheryma whipplei* (2) | blaTEM (218) | Not tested |
| 22T0007717 | Negative | *Haemophilus haemolyticus* (129)  *Aspergillus fumigatus* (2) | blaTEM (3) | Not tested |
| 22T000619 | Negative | *Klebsiella pneumoniae* (8)  *Staphylococcus aureus* (5) | blaTEM (8) | Not tested |
| 22T0016634 | Negative | *Proteus mirabilis* (35)  *Streptococcus pneumoniae* (14) | sul2 (14)  blaTEM (14)  sul1 (13)  tet(B) (4)  aac(3)-IId (11) | Not tested |
| 22T0016636 | Negative | *Proteus mirabilis* (14) | sul2 (5)  blaTEM (4)  sul1 (11)  tet(B) (14)  aac(3)-IId (8) | Not tested |
| 22T0016444 | Negative | *Mycoplasma pneumoniae* (1030) | 98.67% similarity with 23S rRNA | Not tested |
| 22T006314 | Negative | *Pseudomonas aeruginosa* (4726)  *Aspergillus quadrilineatus* (1485) | aph(3')-VI (1)  aac(6')-Ib-G (27)  aac(6')-IId (2)  aac(6')-Ib' (3)  aac(6')-Ib11 (1)  ant(3'')-Ij (2)  aac(6')-30 (2)  aac(6')-Ib-AGKT (1)  aac(6')-Ib-AKT (1)  aac(3)-IIe (1)  blaTEM (2) | Not tested |
| 22T0016115 | Negative | *Haemophilus influenzae* (1245)  *Proteus mirabilis* (55) | aph(3')-VI(3)  sul1(1)  sul2(19)  aac(6')-Ib-G(32)  blaTEM(560)  aac(6')-Ib-AKT(6)  aac(6')-30(3)  aac(6')-IId(5)  blaKPC(1)  aac(6')-Ib-generic(1)  aac(6')-Ib11(1)  aac(6')-Ib'(4)  ant(3'')-Ij(1) | Not tested |
| 21T009464 | Negative | *Mycobacterium tuberculosis* (2) *Klebsiella pneumoniae* (6) | blaSHV(5)  blaCTX-M(2)  blaTEM(4)  blaKPC(3) | Not tested |
| 22T007385 | Negative | *Haemophilus haemolyticus* (24)  *Human gammaherpesvirus 4* (59) | blaTEM | Not tested |
| 22T001700 | Negative | *Campylobacter concisus* (77)  *Streptococcus pneumoniae* (9) | blaTEM (1) | Not tested |
| 22T0009087 | Negative | *Haemophilus haemolyticus* (2343)  *Aggregatibacter segnis* (684)  *Staphylococcus aureus* (25)  *Tropheryma whipplei* (9) | blaTEM(2084) | Not tested |
| 22T001699 | Negative | *Staphylococcus aureus* (342)  *Haemophilus haemolyticus* (27)  *Mycoplasma pneumoniae* (22421) | blaTEM(3) | Not tested |
| 22T0010470 | Negative | *Mycobacterium tuberculosis* (138)  *Stenotrophomonas maltophilia* (6500) | blaTEM(7) | Not tested |

Abbreviations: R Resistant, I Intermediate, S Susceptible

No potential pathogens were detected by Nanopore, but commensals were identified by Nanopore in case noted ^#^.


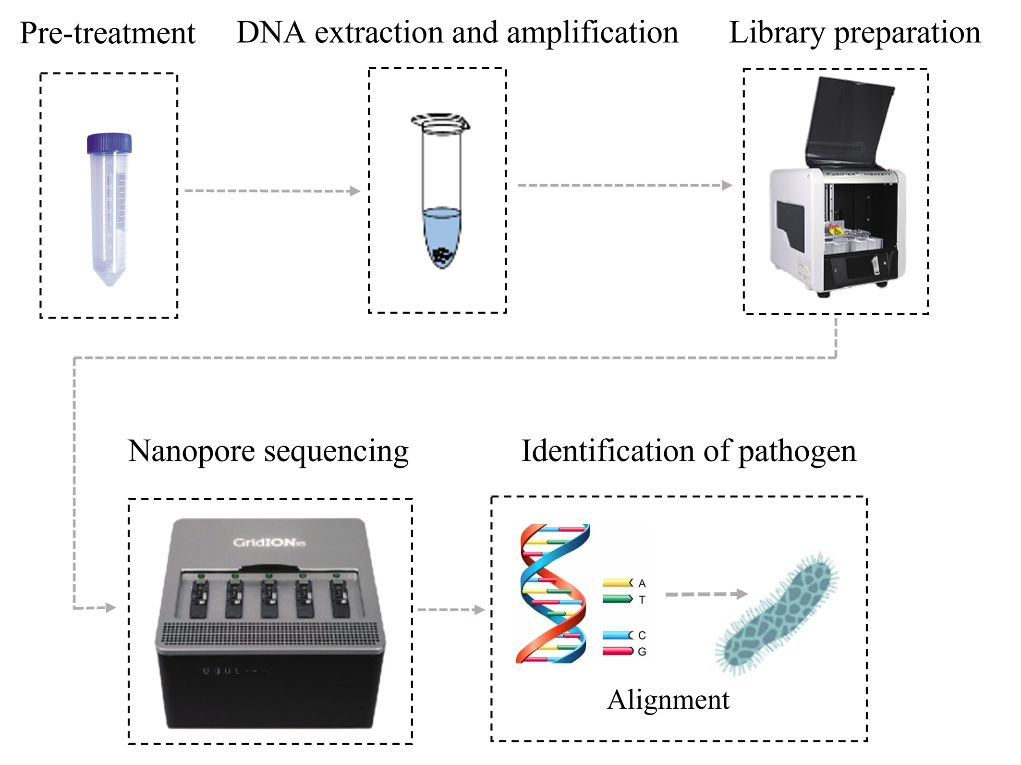


**Supplementary figure 1** Targeted nanopore sequencing workflow.
